# Self-enrolment antenatal health promotion data as an adjunct to maternal clinical information systems in the Western Cape Province of South Africa

**DOI:** 10.1136/bmjgh-2017-000565

**Published:** 2018-04-24

**Authors:** Alexa Heekes, Nicki Tiffin, Pierre Dane, Themba Mutemaringa, Mariette Smith, Nesbert Zinyakatira, Peter Barron, Chris Seebregts, Andrew Boulle

**Affiliations:** 1 Department of Health, Provincial Government of the Western Cape, Cape Town, South Africa; 2 Centre for Infectious Disease Epidemiology andResearch (CIDER), University of Cape Town, Cape Town, South Africa; 3 Jembi Health Systems NPC, Cape Town, South Africa; 4 University of Cape Town, School of Public Health and Family Medicine, Cape Town, South Africa; 5 National Department of Health, Pretoria, South Africa; 6 School of Public Health, University of the Witwatersrand, Johannesburg, South Africa; 7 Wellcome Trust Centre for Infectious DiseasesResearch in Africa (CIDRI-Africa), University of Cape Town, Cape Town, South Africa

**Keywords:** health systems, maternal health, public health

## Abstract

Information systems designed to support health promotion in pregnancy, such as the MomConnect programme, are potential sources of clinical information which can be used to identify pregnancies prospectively and early on. In this paper we demonstrate the feasibility and value of linking records collected through the MomConnect programme, to an emergent province-wide health information exchange in the Western Cape Province of South Africa, which already enumerates pregnancies from a range of other clinical data sources. MomConnect registrations were linked to pregnant women known to the public health services using the limited identifiers collected by MomConnect. Three-quarters of MomConnect registrations could be linked to existing pregnant women, decreasing over time as recording of the national identifier decreased. The MomConnect records were usually the first evidence of pregnancy in pregnancies which were subsequently confirmed by other sources. Those at lower risk of adverse pregnancy outcomes were more likely to register. In some cases, MomConnect was the only evidence of pregnancy for a patient. In addition, the MomConnect records provided gestational age information and new and more recently updated contact numbers to the existing contact registry. The pilot integration of the data in the Western Cape Province of South Africa demonstrates how a client-facing system can augment clinical information systems, especially in contexts where electronic medical records are not widely available.

**Key questions**

**What is already known?**

- Prospective identification of pregnancies enables monitoring of antenatal risk screening and the uptake of interventions in time to impact outcomes.
- Enumerating pregnancies and outcomes at person-level enables a more detailed exploration of maternal and neonatal health services than what is possible from traditionally reported aggregate data.
- The MomConnect programme is an information system designed to support health promotion and is a potential source of clinical information that can be integrated with data traditionally collected by health facilities to create a comprehensive maternal and neonatal care cascade.

**What are the new findings?**

- The pilot integration of MomConnect data with existing clinical data in the Western Cape Province of South Africa demonstrates how a client-facing system can augment clinical information systems.
- Linkage was successful in three-quarters of registrations in spite of the limited identifying data available on which to link.
- Those at lower risk of adverse pregnancy outcomes were more likely to register for MomConnect.

**Key questions**

**What do the new findings imply?**

- Encouraging pregnant women to enrol in health promotion programmes like MomConnect early in their pregnancy may improve their adherence to antenatal care and in turn increase the likelihood of positive birth outcomes. However, there was clear selection bias in those enrolled, cautioning against causal interpretations when looking at programme outcomes.
- The MomConnect programme provided gestational age for a quarter of pregnancies, which is not reliably available at person level from any of the existing clinical information systems in the Western Cape Province of South Africa.
- The lower participation in teenage mothers, who are at higher risk of adverse pregnancy outcomes, indicates that healthcare workers need to focus on providing health promotion initiatives to high-risk groups.

## Introduction

Prospective antenatal identification of the fact and clinical characteristics of pregnancies is an important health information system goal. It enables monitoring of antenatal risk screening and of the uptake of appropriate interventions, with the opportunity to potentially intervene in time to impact outcomes. Full enumeration of pregnancies and associated birth outcomes at person level, even if not resulting in interventions, is further an important part of health system intelligence, enabling much more detailed exploration of the maternal and neonatal services than is possible from aggregate data as traditionally reported through district health information systems.^1^

A mobile health messaging service and helpdesk for South African mothers (MomConnect) was launched as a national initiative in 2014 with the dual intent of providing a platform for health promotion through supportive text messaging to mobile phones of pregnant women and of establishing a registry of pregnancies.^2 3^ Information systems designed to support health promotion through self or facility-based enrolment, or a combination, such as the MomConnect programme in South Africa, are potential sources of clinical information which can be integrated with data from traditional facility-based information systems as part of a comprehensive maternal–neonatal care cascade. Such a cascade can be used for direct service delivery support and for health system intelligence. Data derived from these client-facing information systems, often based on mobile device interfaces, can be of particular value in settings where clinical data are not routinely digitised at health facilities, as is the case in many resource-limited settings where records are paper-based and retained by patients themselves (common for maternity case records) or at facilities.

The aim of this cross-sectional analysis is to demonstrate the feasibility and value of linking records collected through the MomConnect programme for maternal cellphone-based health promotion messaging, to an emergent province-wide health information exchange in the Western Cape Province of South Africa. We describe the characteristics of provincial public sector patients enrolling in MomConnect relative to all pregnant women, determine the linkage success and associations given the limited data available on which to link, estimate the incremental contribution to consolidated clinical and administrative data on pregnancy and explore outcomes for linked patients.

## The Western Cape setting: a province-wide health information exchange

Within the Western Cape Province of South Africa, the Western Cape Government Health (WCGH) Department employs a variety of electronic platforms for routine delivery of healthcare. These platforms include hospital and primary care administrative systems; facility pharmacy and prepackaged chronic drug dispensing systems; and laboratory records. Routinely collected clinical information regarding maternal and child health is restricted to key indicators, such as antenatal visits and immunisations, and is largely reported at aggregate level through the district health information system for the purpose of monitoring and evaluation as well as resource planning.^1^ Although these data are useful for analysing aggregate outcomes, the prospects for patient-level interventions and detailed analyses are limited. The WCGH has established a unique patient identifier which is also used as the folder number within each facility. This has been gradually implemented through a uniform hospital information system in all 52 hospitals over the past two decades and was extended to primary care clinics beginning in 2007. This unique patient identifier enhances the integration of health data in a patient-level health information exchange, the Provincial Health Data Centre (PHDC). The data are uploaded daily from their source systems and linked to individuals in the patient master index (PMI) based on this and other identifiers. In practice, some individuals have multiple folder numbers, for which de-duplication algorithms are used to identify duplicate folder numbers that most likely represent one individual.

Data are further enriched on uptake by identifying a variety of common health episodes such as HIV and pregnancy. Multiple types of evidence are collated to ascertain pregnancy, including laboratory, pharmacy and facility visit data. While some data unequivocally infer pregnancy, such as birth records, inpatient International Statistical Classification of Diseases and Related Health Problems 10th Revision (ICD10) admission and procedure codes that specify pregnancy, antenatal laboratory screening tests (such as for rhesus antibodies) and dispensed drugs specific to termination of pregnancy, others are only suggestive or provide supporting evidence of ongoing care for an already established episode. Examples of supporting evidence include patient encounters at maternity wards or non-specific pregnancy-related dispensed drugs such as folate and iron supplements. The nuances of evidence strength and collation of multiple types of evidence are used to build confidence in the episodes.

In the South African setting, the identification of HIV-infected pregnant women not yet on antiretroviral therapy (ART) and of women who have not been tested for HIV are important risks, in addition to traditional obstetric risks such as maternal age, existing medical conditions and comorbidities, and conditions of pregnancy such as high parity, gestational diabetes and eclampsia.^4^ HIV status is determined from many different electronic sources, including laboratory tests for HIV, CD4 count and viral load, pharmacy records of dispensing of HIV-specific medication and inclusion in the HIV-specific TIER.Net database.^5^

## Integrating self-enrolment data with clinical information systems

The WCGH receives the provincial complement of the MomConnect data in order to integrate records from the public sector facilities into the PHDC. Although the MomConnect registration process is supposed to take place at fixed facilities facilitated by a healthcare provider, due to the limited identifiers available which do not include a folder number, and in keeping with the notional possibility that the system may in future include registrations that have been completed entirely by patients, the data are treated as coming from a source which is not linked to the PMI and are managed similarly to community-based data sources (discussed below). In addition, MomConnect data are treated as supporting evidence of pregnancy, and as such pregnancy episodes are not created based on MomConnect registrations alone. For each pregnancy episode, all sources of evidence supporting the identification of the pregnancy, as described above, are stored, so pregnancies with and without MomConnect registrations can easily be identified.

## Linking patients from community-based data sources to health facility data

Data from services outside health facilities, for example, from community health workers, may come from individuals not yet encountered at public health facilities. Linking data from these individuals to the PMI must therefore include ongoing retrospective scans of unlinked individuals from community-based services in the event that the individual has subsequently visited a health facility and has been assigned a folder number. Similarly, ongoing linkage of enrolment data from MomConnect requires both prospective and retrospective linking. The unlinked records must be retained because they represent individuals who may subsequently require healthcare. Once they access public sector facilities, information about their retrospective access to community-based or self-enrolment services is material to their longitudinal history of access to services.

Available identifiers for linking MomConnect records to the PHDC PMI were South African national identification number (SA ID), date of birth, mobile phone number and sex. To improve linkage, we used two additional pieces of identifiable information: (1) fact of pregnancy, which was used to search for matches to pregnancy episodes where the registration date was within the pregnancy episode period; and (2) the date and facility of registration, which enabled comparison of details for patients who visited the same facility on the same day. Each identifier was ranked based on how well it would uniquely identify a person in the absence of other information; and identifiers were weighted according to their rank, whereby the lowest ranked identifier has a weight of one and each consecutive identifier is assigned a weight double that of the previous identifier.^6^ Identifiers were ranked from highest to lowest (showing weight of ranking in parentheses): SA ID (64), mobile phone number (32), exact date of birth (16), similar date of birth (8), facility and date of registration (4), fact of pregnancy (2) and sex (1). For the linkage to be valid, an exact match had to be made with one of SA ID, mobile phone number or exact date of birth with matching registration date and facility. Once links were found, further restrictions were applied, namely (1) only SA ID was sufficient alone to infer linkage; other identifiers were not strong enough on their own to define linkage; (2) no linkage was inferred if SA ID records were mismatched; (3) the MomConnect record could only link to one individual in the PHDC PMI and (4) probable and possible links were inferred using the combinations of matched identifiers.

In total, 95.2% of registrations took place at a facility using an electronic platform linked to the unique folder number (table 1). Of the linked records, 9.9% were identified as duplicate records, so that the data represent 65 073 individual enrolees. In total, 73.2% of the MomConnect records could be linked to the PMI and 70.8% were linked with high confidence (table 2). There was a high reliance on the civil identifier—of the linked records, the vast majority of records (84.1%) linked using the SA ID combined with further identifying data where available. However, when the completeness of this field declined in later years, so too did the proportion of registrations which could be linked to clinical records. The percentage that were successfully linked decreased from 73.2% in 2014 to 69.8% in 2017, aligned with a decrease in the percentage of registrations with valid SA IDs from 80.7% in 2014 to 63.5% in 2017. All South Africans are issued an SA ID number at birth; however, it is only possible to receive an ID document at 16 years of age. Non-South Africans only receive an ID number once they have attained permanent residence. In the health system, the SA ID is not required to access health services, and as such in the PHDC PMI less than half of individuals have a valid SA ID recorded. While the SA ID number is not essential for the health promotion service, in the absence of other identifiers such as the folder number, the importance of this field needs to be emphasised in the registration workflow. Similarly, the accurate collection of the SA ID in public health facilities should be encouraged as it will improve linkage to other health services that do not have access to the PMI.

**Table 1:** Characteristics of MomConnect registrations in the Western Cape Province 2014–2017

	Total	2014	2015	2016	2017
Total registrations	98 734	2639	28 344	45 951	21 800
Excluded* registrations	991 (1.0%)	106 (4.0%)	509 (1.8%)	332 (0.7%)	44 (0.2%)
SA ID					
Valid SA ID	68 704 (69.6%)	2130 (80.7%)	21 118 (74.5%)	31 605 (68.8%)	13 851 (63.5%)
No valid SA ID provided	30 030 (30.4%)	509 (19.3%)	7226 (25.5%)	14 346 (31.2%)	7949 (26.5%)
Facility type					
National central hospital	1057 (1.1%)	–	322 (1.1%)	617 (1.3%)	118 (0.5%)
Regional hospital	480 (0.5%)	2 (0.1%)	321 (1.1%)	114 (0.3%)	43 (0.2%)
District hospital	2249 (2.3%)	51 (1.9%)	798 (2.8%)	897 (2.0%)	503 (2.3%)
Clinic	91 880 (93.1%)	2350 (89.1%)	25 739 (90.8%)	43 122 (93.8%)	20 669 (94.8%)
Satellite clinic	1488 (1.5%)	54 (2.1%)	491 (1.7%)	639 (1.4%)	304 (1.4%)
Mobile service	589 (0.6%)	76 (2.9%)	164 (0.6%)	230 (0.5%)	119 (0.5%)
Facility authority					
Provincial government	89 291 (90.4%)	2479 (93.9%)	26 568 (93.7%)	41 295 (89.9%)	18 949 (86.9%)
City of cape town	8452 (8.6%)	54 (2.1%)	1 267 (4.5%)	4324 (9.4%)	2807 (12.9%)
PMI site†					
PMI site	94 027 (95.2%)	2380 (90.2%)	26 780 (94.5%)	44 288 (96.4%)	20 579 (94.4%)
Non-PMI site	3716 (3.8%)	153 (5.8%)	1 055 (3.7%)	1331 (2.9%)	1177 (5.4%)
Metro/outside metro					
Metro	56 656 (57.4%)	1066 (40.39%)	16 675 (58.8%)	27 012 (58.8%)	11 903 (54.6%)
Outside of the metro	42 078 (42.6%)	1573 (59.61%)	11 669 (41.2%)	18 939 (41.2%)	9897 (45.4%)
*Age category at registration (years)*					
Median (IQR) age	26.4 (22.3–31.3)				
<20	11 545 (11.7%)	199 (7.5%)	3354 (11.8%)	5396 (11.7%)	2596 (11.9%)
20–24	28 426 (28.8%)	698 (26.5%)	8388 (29.6%)	12 929 (28.1%)	6411 (29.4%)
25–29	26 574 (26.9%)	658 (24.9%)	7737 (27.3%)	12 197 (26.5%)	5982 (27.4%)
30–34	18 026 (18.3%)	393 (14.9%)	4987 (17.6%)	8458 (18.4%)	4188 (19.2%)
35–39	8160 (8.3%)	148 (5.6%)	2108 (7.4%)	3840 (8.4%)	2064 (9.5%)
40–44	1851 (1.9%)	33 (1.3%)	503 (1.8%)	903 (2.0%)	412 (1.9%)
>44	140 (0.1%)	–	46 (0.2%)	68 (0.1%)	26 (0.1%)
Data not available	3021 (3.1%)	404 (15.3%)	712 (2.5%)	1828 (4.0%)	77 (0.4%)
*Gestational age at registration (weeks)*					
Median (IQR)	20 (12.9–28.3)				
0–13	27 580 (27.9%)	280 (10.6%)	6240 (22.0%)	13 757 (29.9%)	7303 (33.5%)
14–19	19 776 (20.0%)	250 (9.5%)	4895 (17.3%)	9836 (21.4%)	4795 (22.0%)
20–31	32 014 (32.4%)	822 (31.2%)	9864 (34.8%)	14 538 (31.6%)	6790 (31.2%)
≥32	16 410 (16.6%)	733 (27.8%)	6349 (22.4%)	6725 (14.6%)	2603 (11.9%)
Registered after EDD	1319 (1.3%)	34 (1.3%)	419 (1.5%)	656 (1.4%)	210 (1.0%)
>40 before EDD	232 (0.2%)	3 (0.1%)	67 (0.2%)	107 (0.2%)	55 (0.3%)
Invalid EDD	412 (0.4%)	411 (15.6%)	1 (0%)	–	–

*Registrations from non-public sector health facilities were excluded.

†PMI site: site assigning and using Clinicom folder numbers.

EDD, estimated delivery date; PMI, Patient Master Index; SA ID, South African national identification number.

**Table 2:** Linkage of MomConnect enrolees to other routine clinical data on pregnancies

	Total	2014	2015	2016	2017
Total records	97 743	2639	28 344	45 951	21 800
Total linked records	71 512 (73.2%)	1984 (75.2%)	21 612 (76.3%)	32 697 (71.2%)	15 219 (69.8%)
Number of individuals	65 073	1828	19 678	29 826	13 741
Duplicate records (%)	9.9	8.7	9.7	9.8	10.6
Probable linkages*	69 217 (70.8%)	1934 (73.3%)	21 133 (74.6%)	31 764 (69.1%)	14 386 (66.0%)
Probable linkage categories					
SA ID, other evidence	60 142 (84.1%)	1914 (96.5%)	18 641 (86.3%)	27 599 (84.4%)	11 988 (78.8%)
DOB, pregnancy, mobile phone	2126 (3.0%)	8 (0.4%)	728 (3.4%)	945 (2.9%)	445 (2.9%)
DOB, pregnancy, mobile phone, encounter	1483 (2.1%)	2 (0.1%)	542 (2.5%)	650 (2.0%)	289 (1.9%)
DOB, pregnancy, encounter	4024 (5.6%)	4 (0.2%)	911 (4.22%)	2063 (6.3%)	1046 (6.9%)
DOB, mobile phone	785 (1.1%)	3 (0.2%)	142 (0.7%)	280 (0.9%)	360 (2.4%)
DOB, mobile phone, encounter	376 (0.5%)	–	80 (0.4%)	106 (0.3%)	190 (1.3%)
YOB, pregnancy, mobile phone	159 (0.2%)	1 (0.1%)	58 (0.3%)	70 (0.2%)	30 (0.2%)
YOB, pregnancy, mobile phone, encounter	97 (0.1%)	2 (0.1%)	28 (0.1%)	42 (0.1%)	25 (0.2%)
YOB, contact, encounter	24 (0.0%)	–	3 (0.0%)	8 (0.0%)	13 (0.1%)
Possible linkage categories					
DOB, encounter, sex	1189 (1.7%)	3 (0.2%)	178 (0.8%)	333 (1.0%)	675 (4.4%)
Mobile phone, pregnancy	527 (0.7%)	19 (1.0%)	136 (0.6%)	309 (1.0%)	63 (0.4%)
Mobile phone, encounter, pregnancy	352 (0.5%)	12 (0.6%)	116 (0.5%)	196 (0.6%)	28 (0.2%)
Mobile phone, encounter	86 (0.1%)	15 (0.8%)	20 (0.1%)	37 (0.1%)	14 (0.1%)
Mobile phone, YOB	79 (0.1%)	1 (0.1%)	16 (0.1%)	34 (0.1%)	28 (0.2%)
DOB, encounter	63 (0.1%)	–	13 (0.1%)	25 (0.1%)	25 (0.2%)
Facility type					
National central hospital	977 (92.4%)	–	306 (95.0%)	557 (90.3%)	114 (96.6%)
Regional hospital	311 (64.8%)	2 (100%)	227 (70.7%)	70 (61.4%)	12 (27.9%)
District hospital	1511 (67.2%)	34 (66.7%)	613 (76.8%)	598 (66.7%)	266 (52.9%)
Clinic	67 366 (73.4%)	1857 (79.0%)	20 037 (77.9%)	30 959 (71.8%)	14 513 (70.5%)
Satellite clinic	974 (65.5%)	41 (75.9%)	343 (69.9%)	406 (63.5%)	184 (60.5%)
Mobile service	288 (48.9%)	50 (65.8%)	86 (52.4%)	107 (46.5%)	45 (37.8%)
Facility authority					
Provincial government	65 922 (73.9%)	1946 (78.5%)	20 702 (77.9%)	29 930 (72.5%)	13 414 (70.8%)
City of Cape Town	520 (65.3%)	38 (70.4%)	910 (71.8%)	2767 (64.0%)	1805 (64.3%)
PMI site†					
PMI site	69 109 (73.5%)	1887 (79.3%)	20 938 (78.2%)	31 870 (72.0%)	14 414 (70.0%)
Non-PMI site	2403 (64.7%)	97 (63.4%)	674 (63.9%)	827 (62.1%)	805 (68.4%)
Metro/outside metro					
Metro	43 104 (76.1%)	815 (76.5%)	13 065 (78.4%)	20 302 (75.2%)	8922 (75.0%)
Outside of the metro	28 408 (67.5%)	1169 (74.3%)	8547 (73.3%)	12 395 (65.5%)	6297 (63.6%)
Age category at registration (years)					
Median (IQR) age	26.7 (22.8–31.5)				
<20	6958 (60.3%)	167 (83.9%)	2179 (65.0%)	3207 (59.4%)	1405 (54.1%)
20–24	21 250 (74.8%)	618 (88.5%)	6734 (80.3%)	9483 (73.4%)	4415 (68.9%)
25–29	20 519 (77.2%)	612 (93.0%)	6283 (81.2%)	9249 (75.8%)	4375 (73.1%)
30–34	14 185 (78.7%)	366 (93.1%)	4109 (82.4%)	6621 (78.3%)	3089 (73.8%)
35–39	6496 (79.6%)	145 (98.0%)	1711 (81.2%)	3034 (79.0%)	1606 (77.8%)
40–44	1471 (79.5%)	31 (93.9%)	413 (82.1%)	727 (80.5%)	300 (72.8%)
>44	97 (69.3%)	–	29 (63.0%)	48 (70.6%)	20 (76.9%)
Data not available	536 (17.7%)	45 (11.1%)	154 (21.6%)	328 (17.9%)	9 (11.7%)
Gestational age at registration (weeks)					
Median (IQR)	19.9 (12.9–28.1)				
0–13	20 529 (74.4%)	256 (91.4%)	4925 (78.9%)	10 142 (73.7%)	5206 (71.3%)
14–19	14 721 (74.4%)	230 (92.0%)	3862 (78.9%)	7167 (72.9%)	3462 (72.2%)
20– 31	23 343 (72.9%)	759 (92.3%)	7578 (78.6%)	10 275 (70.7%)	4731 (60.7%)
≥32	11 879 (72.4%)	663 (90.5%)	4887 (77.0%)	4641 (69.0%)	1688 (64.9%)
Registered after EDD	822 (62.3%)	30 (88.2%)	310 (74.0%)	395 (60.2%)	87 (41.4%)
>40 before EDD	175 (75.4%)	3 (100%)	50 (74.6%)	77 (72.0%)	45 (81.8%)
Invalid EDD	43 (10.4%)	43 (10.5%)	0 (0%)	–	–

*Probable linkage means that the linkage is highly likely to be correct. Possible linkage means that the linkage is fairly likely to be correct.

†PMI site: site assigning and using Clinicom folder numbers.

DOB, date of birth; EDD, estimated delivery date; PMI, Patient Master Index; SA ID, South African national identification number; YOB, year of birth.

It was encouraging that a high proportion of the MomConnect records could be linked to known pregnancies from clinical data sources. For those without the SA ID linkage, further linkage could be established using combinations of date of birth, fact of pregnancy, mobile phone number and the registration visit to a facility on a specific date matching a visit record in the PHDC. The combination of encounter, pregnancy and date of birth as identifiers was the second highest means of linkage at 5.6% overall. Additional identifiers, including the folder number and names, would further assist linkage to clinical records. There were nevertheless some useful learnings on linkage inference in the context of sparse identifying data, including the value of limiting match sets by location, date and health condition—that is, only trying to link to pregnant women who visited the same facility on the same day—and rejecting links where there is more than one possible match.

In addition, although the PHDC had existing contact numbers for the vast majority of the individuals registered in MomConnect, the MomConnect records provided several new and more recently updated contact numbers to the existing contact registry. Of all the individuals registered in MomConnect, 89.7% of them have a contact number in the PMI. However only 46.9% have the same contact number in the PMI and MomConnect, and 63.1% have an additional contact to the PMI in MomConnect. Overall 42 553 additional contact numbers pertaining to 41 069 individuals could be added to the PMI from MomConnect. 96.9% of individuals had one mobile number recorded in MomConnect, 2.8% had two mobile numbers recorded and 0.04% had three or more contact numbers due to multiple registrations.

## Associations with registration

The multivariable analysis of associations with MomConnect registration (table 3), where the registrations could be linked as described above, demonstrated that teenagers and older women, patients with the first evidence of pregnancy at a location other than a primary care clinic and patients in the metropolitan area were all less likely to register. The temporal trend towards increased registration was also evident when pregnancies ascertained in 2016 were compared with 2015, the two years with complete data available.

**Table 3:** Multivariable analysis of associations with MomConnect registration

Factor	Univariable model	Multivariable model
	OR (95% CI)	OR (95% CI)
HIV status		
Positive	1.00 (0.98 to 1.02)	0.80 (0.78 to 0.82)
Age category (years)		
<20	0.71 (0.68 to 0.73)	0.63 (0.62 to 0.66)
25–29	1.00 (ref)	1.00 (ref)
20–24	1.07 (1.05 to 1.09)	1.01 (0.98 to 1.03)
30–34	0.89 (0.87 to 0.91)	0.82 (0.80 to 0.85)
35–39	0.77 (0.75 to 0.80)	0.78 (0.75 to 0.81)
40–44	0.51 (0.48 to 0.54)	0.47 (0.44 to 0.51)
>44	0.12 (0.10 to 0.14)	0.05 (0.04 to 0.06)
Facility type		
Primary healthcare*	1.00 (ref)	1.00 (ref)
MOU	0.20 (0.20 to 0.20)	0.39 (0.38 to 0.39)
Regional hospital	0.03 (0.03 to 0.03)	0.02 (0.02 to 0.02)
District hospital	0.04 (0.04 to 0.04)	0.02 (0.02 to 0.02)
National central hospital	0.03 (0.03 to 0.04)	0.06 (0.06 to 0.06)
Pregnancy year†		
2014	0.13 (0.12 to 0.13)	0.12 (0.12 to 0.13)
2015	1.00 (ref)	1.00 (ref)
2016	1.58 (1.54 to 1.60)	1.63 (1.59 to 1.67)
2017	0.99 (0.96 to 1.01)	0.87 (0.85 to 0.90)
Metro/outside metro		
Outside the metro	1.64 (1.61 to 1.67)	4.88 (4.74 to 5.03)

*The primary healthcare facility type combines clinic, satellite service and mobile service in table 1 and excludes maternity and obstetric units (MOUs).

†Data are incomplete for 2014 and 2017.

It was not surprising that patients presenting outside of routine primary care would be less likely to register, given that those presenting for the first time at hospitals would likely have pregnancy-associated risk factors and be in larger clerical environments oriented to referral rather than first booking services. Data on parity were not available to determine if the decline in registration in older women was related to less subjective need for pregnancy advice. The lower participation in teenage mothers aligns with lower participation and adherence across a range of health conditions and services in this age group.^7–9^

## Health service contribution of MomConnect data

The WCGH has elected so far not to delineate pregnancies based just on MomConnect data, in case there are false registrations. However, MomConnect data are used to strengthen inference around pregnancy where there are multiple data points which are deemed to provide moderate confidence of a pregnancy episode. The current analysis has demonstrated that a meaningful proportion of pregnancies might be identified by MomConnect and no other systems, even where a high proportion of pregnancies are appearing in or can be inferred from other electronic clinical systems. Ascertained public sector pregnancies (table 4) were approximately 118 000 in 2015 and 2016 (year of pregnancy reflects the year in which the first evidence of pregnancy falls). Of these, the proportion that had MomConnect registrations as an evidence of pregnancy was 18.1% and 25.9%, respectively. Of the MomConnect registrations that linked to existing pregnancies, 64.5%–70.5% have outcome data between 2014 and 2016, broadly similar to the proportions for pregnancies without MomConnect registrations. Outcome data are still very low for pregnancies first detected in 2017 as many of these pregnancies have not yet reached term. There are a small number of pregnancies that are potentially ascertained only through MomConnect (2986 or an additional 2.5% in 2016), not being evidenced through other clinical data. Of note, in 2017 this figure is the highest, suggesting that at least some of these individuals may yet connect to public sector healthcare as their pregnancy progresses.

**Table 4:** Contribution of MomConnect data to public sector pregnancy enumeration

	2014	2015	2016	2017
Total public sector-ascertained pregnancies	108 336	118 522	118 943	72 402
Linked MomConnect registration	2894 (2.7%)	21 404 (18.1%)	30 758 (25.9%)	13 414 (18.5%)
Pregnancies without MomConnect registration	105 442 (97.3%)	97 118 (81.9%)	88 185 (74.1%)	58 988 (81.5%)
Only MomConnect registration as evidence*	537	2380	2986	3955
Total pregnancies with data on outcomes	76 101 (70.3%)	82 455 (69.6%)	81 047 (68.1%)	28 821 (39.8%)
MomConnect registrations with data on outcomes	1867 (64.5%)	14 977 (70.0%)	21 688 (70.5%)	3911 (29.2%)
Pregnancies without MomConnect registration and with data on outcomes	74 234 (70.4%)	67 478 (69.5%)	59 359 (67.3%)	24 910 (42.3%)

*Pregnancies with only MomConnect registration as evidence are not currently incorporated into the pregnancy episodes and therefore do not form part of the total count. These totals however reflect the number of additional pregnancies which could potentially be ascertained through the addition of MomConnect data.

In order to have data about pregnancies available antenatally, there is currently a high reliance on clerical and laboratory data (eg, Rhesus antibody testing), which do not include clinical parameters such as gestational age. The MomConnect programme records the estimated delivery date, from which gestational age at registration was calculated from the difference between the estimated delivery date and registration date, assuming an average gestational period of 40 weeks. Overall, 27.9% of MomConnect registrations between 2014 and 2017 were at a gestational age between 0 and 13 weeks, increasing to nearly a third of all MomConnect registrations in recent years (table 1). In 2016, gestational age was prospectively available in a quarter of pregnancies as a result of the MomConnect programme, a tangible clinical contribution from the initiative. As MomConnect becomes more widely used, the contribution of gestational age could have immense value for obstetric risk management. For patients with pre-existing chronic conditions such as diabetes and hypertension, knowledge of pregnancy early on could be communicated to health facilities to ensure appropriate antenatal care is provided. From a public health research perspective, knowledge of gestational age could, for example, enable more detailed studies of teratogenic effects of medications during pregnancy. This is a demonstration of how a client-facing system can augment clinical information systems, especially in contexts where electronic medical records are not widely available, and there is limited digitisation and updating of clinical and clerical data.

## Linking to pregnancy outcomes

The earliest full year for which data are available and most likely to have outcome data is 2015. Analysis of pregnancy outcomes in 2015 (table 5) shows that mothers enrolling in MomConnect have fewer adverse pregnancy outcomes. Of those mothers with an available birth outcome, 97.5% of those enrolled in MomConnect had a live birth compared with 89.7% of those who were not enrolled in MomConnect. The proportion of mothers with confirmed HIV infection comparing those with and without MomConnect registration (table 5) was 19.6% and 22.0%, respectively, with slight differences in when this status was first known to the WCGH, the MomConnect mothers being less likely to have been previously identified as HIV-infected. While the results of point-of-care HIV screening tests are not captured electronically at present, we have assumed that ‘unknown’ status is most likely HIV negative status based on the knowledge that in the Western Cape the vast majority of pregnant women are screened for HIV.^10^

**Table 5:** Illustrative comparison of birth outcomes and HIV status in public sector pregnancies (2015) with and without MomConnect registration

	Total Western Cape	Without MomConnect registration	With MomConnect registration
Birth outcomes (n)	82 455	67 478	14 977
Live birth	75 154 (91.1%)	60 544 (89.7%)	14 610 (97.5%)
Stillbirth	1774 (2.2%)	1571 (2.3%)	203 (1.4%)
Miscarriage	2422 (2.9%)	2310 (3.4%)	112 (0.7%)
Termination	3105 (3.8%)	3050 (4.5%)	55 (0.4%)
HIV-positive status (n)	118 522	97 118	21 404
Unknown*	84 539 (71.3%)	68 664 (70.7%)	15 875 (74.2%)
Negative	8433 (7.1%)	71 000 (7.3%)	1333 (6.2%)
Positive	25 550 (21.6%)	21 354 (22.0%)	4196 (19.6%)
Categories for HIV-positive pregnant women			
ART prior to pregnancy	15 622 (61.1%)	13 359 (62.6%)	2263 (53.9%)
ART during pregnancy	5087 (19.9%)	3619 (16.9%)	1468 (35.0%)
Positive prior to pregnancy, no antenatal or postnatal evidence of ART	1725 (6.8%)	1555 (7.3%)	170 (4.1%)
Positive prior to pregnancy, started ART postnatally	667 (2.6%)	638 (3.0%)	29 (0.7%)
Positive during pregnancy, no antenatal or postnatal evidence of ART	497 (1.9%)	453 (2.1%)	44 (1.0%)
Positive during pregnancy, started ART postnatally	561 (2.2%)	527 (2.5%)	34 (0.8%)
Positive after pregnancy, no antenatal or postnatal evidence of ART	738 (2.9%)	636 (3.0%)	102 (2.4%)
Positive after pregnancy, started ART postnatally	653 (2.6%)	567 (2.7%)	86 (2.0%)

*HIV testing is predominantly done through point-of-care tests with the results not captured digitally. Since the overwhelming majority of pregnant women are tested for HIV antenatally and those with HIV are readily identifiable from other data sources, unknown HIV status usually implies being HIV-uninfected.

ART, antiretroviral therapy.

While it is tempting to infer intervention effects from the differences in pregnancy outcomes between MomConnect registrations and other pregnancies, these differences are almost certainly the result of selection bias. For example, the fewer terminations of pregnancy among women registered in MomConnect are most likely because women intending to terminate their pregnancy would be less likely to register. Those at lower risk of adverse pregnancy outcomes were more likely to register, reflecting this substantial selection bias as to who registers with the programme. A further caution is that in spite of the reporting in this analysis of a high proportion of registrations being linked, over a quarter did not link, potentially introducing further selection bias with respect to which MomConnect registrations are being compared with other pregnancies. Nevertheless, the ability to link to clinical outcomes is an important prerequisite for long-term evaluation of health promotion activities, given appropriate study designs.

## Data governance and management

MomConnect data represent a hybrid data source crossing self-enrolment for health promotion and clinician-mediated facility-based enrolment. In order to use these data for clinical purposes, it is important that consenting procedures are clear on the dual intent of the registration and are verified directly with the participant after registration if the registration has been completed on their behalf. In this analysis, only participants who registered at public health facilities were included. However, with appropriate consent the analysis could be extended to include private registrations which will be a valuable piece of information for the individuals who do not seek public antenatal care but choose to deliver in public facilities. An option to withdraw at any point is also required and is currently provisioned for by the system.

When using self-enrolment data which are not facility-mediated or could come from outside the jurisdiction, and consent is given for use, these data need to be retained in community rather than patient databases to accommodate people who are not patients of the health system, but who might subsequently become health system users. For many community-based health services, this a model which enables linkage to care to be tracked for people referred from the community to health services. Similarly for self-enrolment services, such as those for health promotion, a link to formal health services can be retrospectively confirmed when the patients register at the formal health services if the historic unlinked data are retained separately in a community database.

## Conclusions

This analysis has demonstrated that a substantial proportion of pregnant women known to the public health services in the Western Cape did register with the MomConnect service, increasing over time since the launch of the initiative, although to date there are more who have not registered than who have. For those who did register, in spite of very limited identifying information available from the registration process, nearly three-quarters could be linked to pregnant women known to the public health services through a combination of clinical data sources. The MomConnect records were usually the first evidence of pregnancy in pregnancies which were subsequently confirmed by other sources and contributed data on gestational age and additional contact details. If the data were treated as reliable evidence of pregnancy without corroboration, there are a number of pregnancies which could have been ascertained only through MomConnect and for which there was no other evidence in the clinical information systems.

The MomConnect initiative has a clear contribution to make as part of an integrated information system in support of clinical services. The pilot integration of the data in the Western Cape Province of South Africa has demonstrated feasibility and value, and is a model for how hybrid information systems which are both client-facing and intended for registering clinical events, can be incorporated into routine clinical information systems.
